# Disparities in Seasonal Influenza Vaccination in Europe

**DOI:** 10.1002/iid3.70186

**Published:** 2025-07-08

**Authors:** Katinka M. Giezeman‐Smits, Bram Palache, Gerrit A. van Essen, Miloš Jeseňák, Enrique Castro‐Sánchez, Anna Elisabeth Steinberg, Joris van Vugt

**Affiliations:** ^1^ Viatris Turmstrasse 24 Steinhausen Switzerland; ^2^ FluPal Consultancy B.V. Bourgondischelaan 33 Amstelveen the Netherlands; ^3^ Dutch Influenza Foundation Paladijnenweg 30 Amersfoort the Netherlands; ^4^ Comenius University in Bratislava, University Hospital in Martin Slovakia; ^5^ Brunel University London, College of Business, Arts and Social Sciences Uxbridge UK; ^6^ Imperial College London NIHR Health Protection Research Unit in Healthcare‐Associated Infection and Antimicrobial Resistance London UK; ^7^ Global Health Unit, University of the Balearic Islands Palma Spain; ^8^ Universidad Internacional de Valencia Valencia Spain; ^9^ Viatris Benzstraße 1 Bad Homburg vor der Höhe Germany; ^10^ Viatris Krijgsman 20 Amstelveen the Netherlands

**Keywords:** comorbidity, ethnic disparity, inequality, influenza, seasonal influenza vaccination, vaccine access, vaccine hesitancy

## Abstract

**Background:**

Seasonal influenza remains a major public health challenge in Europe, associated with high morbidity, mortality, and socioeconomic burden. Despite the proven efficacy and cost‐effectiveness of vaccination, coverage rates vary substantially across European countries and population groups, often falling short of the World Health Organization's target of 75% for older adults.

**Objective:**

This narrative review aims to identify and explore disparities in seasonal influenza vaccination coverage across European countries and among different population subgroups. It also seeks to examine the underlying causes of these disparities and propose actionable strategies to improve equity in vaccine uptake.

**Methods:**

A literature search was conducted using the PubMed database for English‐language articles published within the last 5 years. Keywords included: comorbidity, disparity, Europe, inequality, influenza, knowledge, ethnic disparity, seasonal influenza vaccination, socioeconomic disparity, vaccination coverage, and vaccine hesitancy. Additional references were identified from the retrieved articles.

**Results:**

Widespread disparities in influenza vaccination were observed across various demographic and professional groups. These included disparities by geographic location, ethnicity, socioeconomic status, sex and gender, age, comorbidities, and occupation—particularly among healthcare professionals. Contributing factors included limited knowledge or education, negative attitudes or behaviors toward vaccination, vaccine hesitancy and fatigue, and restricted access to vaccination services. Structural barriers and institutional trust issues also played key roles.

**Conclusions:**

Addressing disparities in influenza vaccination coverage in Europe requires multi‐level, stakeholder‐specific strategies. These should include education campaigns, improved access through alternative delivery settings (e.g., pharmacies, schools), targeted communication to high‐risk and underserved populations, and systemic changes to support healthcare providers. Tackling these issues will help reduce preventable morbidity and mortality, enhance herd immunity, and foster healthier ageing across European populations.

**Abbreviations**
EAA: European Economic Area
ECDC: European Centre for Disease Prevention and Control
EEA: European Economic Area
EU: European Union
GP: General Practitioner
HIV: Human Immunodeficiency Virus
HR: Hazard Ratio
ICU: Intensive Care Unit
NIHR: National Institute for Health Research
OR: Odds Ratio
UK: United Kingdom
USA: United States of America
WHO: World Health Organization

## 1 Background

Influenza is a cause of significant morbidity and mortality, and is associated with important healthcare, economic, and social burden [1, 2]. Healthcare systems are under great pressure to manage the continuous threat of influenza because of the evolving nature of the causative virus [1]. Furthermore, the numerous mild‐to‐moderate cases of influenza can result in absenteeism (from work or school), reduced productivity and inability to care for dependants [3], as well as putting pressure and costs on primary and secondary healthcare and social care services [2]. Risk groups for influenza include populations at particular risk of developing severe disease resulting in hospitalisation or death, such as older adults and individuals with comorbidities, pregnant women, and those at increased risk of exposure to or transmission of influenza virus, such as healthcare workers [4].

The European Centre for Disease Prevention and Control (ECDC) estimates that seasonal influenza causes up to 50 million symptomatic infections in the European Union/European Economic Area (EU/EEA) each year, and 15,000–70,000 European citizens die annually from influenza‐related causes [2]. Influenza‐associated deaths have increased over time in the EU/EAA [5, 6]. Mortality attributed to influenza across the EU is not homogeneously distributed. In 2020, the highest total standardised death rate for influenza was reported in Austria (3.3/100,000 inhabitants) and the lowest in Slovakia (0.1/100,000) [6]. Poland, Hungary, and Romania also reported low rates (< 0.5/100,000) [6]. Influenza mortality was also higher among those aged over 65 years (from 14.7 deaths/100,000 over 65 in Austria, to 0.3/100,000 over 65 in Slovakia) than in those aged under 65 (0.6 deaths/100,000 under 65 in Estonia and Austria, to 0.0/100,000 under 65 in Malta) [6]. Influenza represents the greatest burden of disease and disability‐adjusted life years (DALYs) out of 31 selected diseases in the EU/EEA (30% of the total burden) [7].

Influenza vaccination is the fundamental public health intervention currently available to reduce the morbidity and mortality associated with influenza [8, 9]. Seasonal influenza vaccination in adults is an effective strategy to prevent one in four intensive care unit (ICU) admissions and one in three deaths caused by influenza [10, 11]. Moderate overall protection (53%) against influenza‐associated hospitalisation in children has also been reported [12]. In addition, influenza vaccination reduced the severity of disease even in patients in whom it did not prevent infection and influenza‐associated hospitalisation [13]. Seasonal influenza vaccination has been reported to be a cost‐effective measure in research conducted in different populations across the EU/EAA [14, 15, 16, 17, 18, 19, 20, 21, 22, 23, 24, 25].

Findings such as the reduced morbidity and mortality of influenza and the cost‐effectiveness of seasonal influenza vaccination programmes support the widespread administration of annual influenza vaccination in Europe. However, influenza vaccination coverage rates vary considerably across Europe and, despite an increased intention to vaccinate against influenza internationally during the COVID‐19 pandemic [26], influenza vaccination rates have dropped dramatically compared with pre‐COVID‐19 pandemic levels in some European countries where rates were quite high [27]. Seasonal influenza vaccination rates are currently below the target of 75% recommended by the World Health Organization (WHO) for older adults [28, 29], with coverage rates below 20% in some countries [11, 30]. The WHO recommends that all countries should consider implementing seasonal influenza immunisation programmes, particularly for at‐risk populations [4].

In 2009, the Recommendation by the Council of the European Union set an objective for EU Member States to achieve a 75% vaccination coverage rate with the seasonal influenza vaccine by the 2014–2015 influenza season in specific target populations, such as older individuals, those at risk of more severe disease, and healthcare workers [31]. The ECDC also emphasises that all Europeans who are recommended to have the influenza vaccine should get vaccinated, particularly those at higher risk of serious influenza complications [32].

The indirect effects, or herd immunity, associated with influenza vaccine have long been recognised and accepted [33, 34]. The target vaccination coverage proposed in Europe (75% in elderly and high‐risk individuals) and the registered vaccination coverage have been reported to be insufficient to establish herd immunity against influenza viruses [35].

The aim of this narrative review is to identify the disparities in seasonal influenza vaccination between different European countries and populations, and to discuss the potential reasons for these disparities. A further objective is to explore possible strategies to address these disparities, with the goal of achieving more consistent influenza vaccination coverage rates across the continent. Information for this review was derived from a literature search on the PubMed database for English language articles published in the last 5 years, and references therein, using relevant keywords, including comorbidity, disparity, Europe, inequality, influenza, knowledge, ethnic disparity, seasonal influenza vaccination, socioeconomic disparity, vaccination, vaccination coverage, vaccination rates, vaccine access, and vaccine hesitancy.

### 1.1 Current Disparities in Seasonal Influenza Vaccination

Current disparities in seasonal influenza vaccination include disparities related to geographical location, ethnic group, socioeconomic position, sex and gender, age, or comorbidities. These disparities are considered separately below; however, in reality, many of these disparities are interconnected.

#### 1.1.1 Geographical Disparity in Seasonal Influenza Vaccination

Considering the WHO recommendation that 75% of older adults should be vaccinated against influenza each year, there are significant gaps in influenza immunisation in Europe, and vaccination rates vary over time and between countries in this continent [6, 29]. Data from 2018 to 2019 showed that only one of 28 countries reporting coverage in the WHO European Region achieved that target (Belarus), and only four other countries achieved greater than 60% coverage (Ireland, Portugal, Russian Federation, and the UK) [29]. Vaccination coverage was less than 35% of the older population in half the countries during this period, with the lowest coverage less than 1% [29]. Few countries had mechanisms to monitor vaccination coverage in target groups such as older adults [29].

Data from 2021 confirm that influenza vaccination rates in older populations vary between EU Member States, ranging from the highest rate in Ireland (75.4%), followed by Denmark (75.0%), the Netherlands (72.6%) and Spain (67.7%), to low rates in Slovakia (12.9%), Poland (10%), Bulgaria (8.9%) and Latvia (7.7%) [6]. Overall, just over half (50.8%) of individuals aged at least 65 years in the EU were vaccinated against influenza in 2021 [6].

#### 1.1.2 Disparity in Seasonal Influenza Vaccination Between Different Ethnic Groups

Research into disparities in influenza vaccination between different ethnic populations is primarily conducted in the USA, with Black, Hispanic, and Asian individuals noted to be substantially less likely to receive influenza vaccine than non‐Hispanic White individuals [36, 37, 38, 39, 40]. Disparities in maternal vaccine acceptance among ethnic groups in the USA are well documented, with Black women the least confident in the safety of the maternal influenza vaccine [41]. In addition, Black and Hispanic women were less likely than White women to perceive the risk of acquiring vaccine‐preventable diseases and to trust vaccine information from healthcare providers and public health authorities [41].

In a study of at‐risk adults in England, relative to White patients, Black patients were least likely to be vaccinated (odds ratio [OR]18–64 years: 0.82, and OR65+ years: 0.59), whereas Asian patients among 18–64 year olds were most likely to be vaccinated (OR18–64 years: 1.10) [42]. Another study in England showed that compared to the ‘White British’ group, inequalities in influenza vaccine uptake were most prominent in the groups ‘White and Black Caribbean’ (hazard ratio [HR] 0.63) and ‘White and Black African’ (HR 0.67) [43]. In contrast, uptake was slightly higher than the White British group in the groups ‘other ethnic group’ (HR 1.11) and ‘Bangladeshi’ (HR 1.08) [43]. Lower uptake rates for influenza vaccine were also linked to nonwhite ethnicity among pregnant women in an additional study in England, with the lowest among Black women [44].

In addition, lower seasonal influenza vaccine uptake compared with the host population has been reported in immigrants [45], and certain mobile population groups, such as some Gypsy, Roma, Travellers and migrants [46].

#### 1.1.3 Socioeconomic Disparity in Seasonal Influenza Vaccination

Socioeconomic position is one of the principal determinants of health, and affects an individual's ability to fully comply with preventive measures, including vaccination, and healthcare prescriptions [47]. Socioeconomic deprivation is correlated with low influenza vaccination coverage among groups at risk, such as older adults [42, 47, 48]. Effective targeted interventions to increase influenza vaccination rates should take account of socioeconomic determinants of inequalities in vaccine uptake [45, 49].

Living alone is an overlooked factor in vaccination programmes, particularly in older adults [45]. Higher seasonal influenza vaccine uptake in individuals aged ≥ 60 years from Europe was reported for those not living alone (OR 1.39), with higher income (OR 1.26) and with higher education (OR 1.05) [45]. Lower seasonal influenza vaccine uptake was observed in populations in more deprived areas (risk ratio 0.93) [45].

A study of differences in vaccination coverage, by socioeconomic position, among people aged ≥ 65 years in Italy showed that belonging to families with three or four members was associated with increased coverage rates [49]. In the most deprived group, vaccine uptake was positively associated with the dependency ratio (a measure of how many people depend on the working‐age population for economic support [49, 50].

Vaccine uptake levels in the homeless population have been reported to be less than half those seen among eligible general practitioner (GP) patient groups in a study in England [51]. This finding clearly shows the health inequalities and ‘inverse care law’ that impact this vulnerable population, and highlights the need to strengthen efforts to ensure homeless individuals have access to influenza vaccination [51].

Socioeconomic inequalities in annual seasonal influenza vaccine uptake were exacerbated during the COVID‐19 pandemic, as noted in a study in England, likely due to changes in demand for vaccination, new delivery models, and disruptions to healthcare and schooling [52]. It is crucial to improve understanding of drivers for vaccine uptake inequalities and to address and reverse these growing inequalities [52].

#### 1.1.4 Disparities in Seasonal Influenza Vaccine Uptake Related to Sex and Gender

Biological (sex) differences as well as gender norms, roles, and relations can lead to differences in males and females in terms of acceptance of vaccination [53, 54]. This variability is seldom taken into account in the planning and execution of vaccination strategies [54].

A multi‐site, retrospective chart review study in the USA comprising 1193 patients showed that the rate of influenza vaccination is higher for females than for males who presented for an annual preventive physical examination and who were younger than 75 years old [55]. The study authors stated that the reasons for the difference between males and females in the rate of influenza vaccination are not clear; however, there are likely influences on vaccination rate between sexes based on culture, age, and sex‐based differences in biological response to vaccination [55]. Similarly, in a study of at‐risk adults in England, females were more likely than males to be vaccinated among 18–64 year olds (OR18–64 years: 1.19) [42]. However, conversely, a nationwide cross‐sectional study in Poland showed that males were more often vaccinated than females (OR 1.56) [56].

#### 1.1.5 Disparities in Seasonal Influenza Vaccine Uptake Related to Age

Uptake of influenza vaccine in adults aged 18–64 years is relatively low, even among the older adults in this population (i.e., aged 50–64 years), perhaps indicating a lack of perceived need for vaccination [3]. In a study of at‐risk adults in England, overall seasonal influenza vaccine uptake was 35.3% and 74.0% for individuals aged 18–64 and over 65 years, respectively [42]. A nationwide cross‐sectional study in Poland also showed that people aged over 65 years received influenza vaccination more often than younger counterparts (OR 1.64) [56].

Considering the increased risk of comorbidity and general declining health in older age, extending the prioritisation of influenza vaccination to the 18–64 years population, or at least to individuals aged 50–64 years, may reduce the risk of hospitalisation and death in this large, working‐age population [3].

Vaccinating children and adolescents against seasonal influenza may reduce disease burden in both vaccinated and unvaccinated individuals because of the pivotal role that younger age groups play in the transmission of infection [57]. Furthermore, young children are prone to severe influenza disease [58]. In the Netherlands, GP consultation rates associated with influenza infection were reported to be highest for children aged under 5 years [59].

Several European countries, including the UK and Finland, have initiated immunisation programmes for healthy toddlers and young children [58]. Efforts to improve influenza burden across Europe could include extending vaccination programmes to younger age groups in those countries that have not already done so. The introduction of new childhood influenza vaccination programmes provides an opportunity to further reduce the impact of influenza in the targeted age groups, and in the wider community [8].

#### 1.1.6 Disparities in Seasonal Influenza Vaccine Uptake Related to Comorbidities

Influenza is associated with high levels of hospitalisation and outpatient visits, and places a burden on patients and healthcare systems that is exacerbated by comorbidities [3]. There are few studies on seasonal influenza vaccine uptake among individuals with disabilities or comorbidities, with many of these conducted outside Europe and showing mixed results [60, 61, 62, 63, 64, 65]. For example, in the USA, vaccination rates have been reported to be low for children with neurodevelopmental disorders (52%) or epilepsy (59%), and only one in two physicians appeared to recognise these conditions as high‐risk in the context of influenza [66, 67].

Focussing on Europe, a nationwide population study in France showed that individuals with disabilities were more frequently vaccinated than other at‐risk groups, mainly because of their higher levels of morbidity and healthcare use [60]. In a cross‐sectional study of adults with chronic conditions in Italy, 64.7% of participants were aware that influenza can be prevented with vaccines and that patients with chronic diseases are at higher risk of developing severe complications; however, only 42.1% had received influenza vaccine in the previous season, and 46.9% planned to receive it in the next season [68]. The level of awareness was significantly lower among the elderly (≥ 65 years) and those with a higher self‐reported health [68]. Factors including older age, greater knowledge of vaccine utility and safety, chronic respiratory disease, or taking more drugs were associated with higher likelihood of vaccination in participants [68].

Patients with immune‐mediated inflammatory diseases, such as rheumatoid arthritis, are at increased risk of infection, in part because of the disease itself, but the primary reason is treatment with immunomodulatory or immunosuppressive drugs [69]. However, self‐reported uptake of vaccination against seasonal influenza was only 59% in a study in Denmark of patients with rheumatoid arthritis receiving immunosuppressive drugs [70]. Furthermore, the majority of patients with multiple sclerosis in a cross‐sectional, single‐centre study in Austria did not fulfil vaccination recommendations for 17 different diseases, with only 10% vaccinated against influenza [71]. Influenza vaccination rate was also low (6.7%) in patients with newly diagnosed HIV infection in a cross‐sectional study of vaccination coverage in the Czech Republic [72]. Higher level of education and less advanced HIV infection were associated with higher vaccination rates in this population [72].

Suboptimal influenza vaccine uptake (41.8% in the latest season) was also reported in a Danish study in kidney transplant recipients and patients on the kidney transplant waiting list [73]. Prior influenza vaccine uptake was positively associated with influenza vaccine uptake in the latest season (*p* < 0.001), whereas recommendations given by non‐physicians were negatively associated [73]. In a nationwide cohort study including all patients aged > 18 years with heart failure in Denmark, influenza vaccination coverage ranged from 16% to 54% during the study period [74]. Influenza vaccination was associated with a reduced risk of both all‐cause and cardiovascular death after extensive adjustment for confounders, with frequent vaccination and vaccination earlier in the year associated with larger reductions in risk of death compared with intermittent and late vaccination [74].

Odds of vaccine uptake in a study of at‐risk adults in England were highest among younger patients with diabetes (OR18–64 years: 4.25) and older patients with chronic respiratory disease (OR65+ years: 1.60), whereas they were lowest among morbidly obese patients of all ages (OR18–64 years: 0.68; OR65+ years: 0.97) [42].

#### 1.1.7 Disparities in Seasonal Influenza Vaccination Among Healthcare Professionals

Uptake of seasonal influenza vaccination among healthcare professionals varies considerably across Europe, as shown by the following examples.

Self‐declared vaccination coverage was 34.8% for influenza among healthcare professionals in a national cross‐sectional study in 2019 in France [75]. Higher influenza vaccination coverage was observed in healthcare professionals working in wards where there was a staff vaccination contact person, staff vaccination was organised in the ward, information on influenza vaccines was provided, and/or the ward manager supported the healthcare professional vaccination campaign [75].

A survey by O'Lorcain et al. of hospitals and long‐term/residential healthcare facilities in Ireland during each season from 2011 to 2012 to 2019–2020 showed that influenza vaccine uptake by healthcare professionals increased over the nine seasons [76]. Uptake among healthcare professionals employed in publicly‐funded hospitals increased from 18.1% (2011–2012) to 58.9% (2019–2020), and uptake in publicly‐funded long‐term/residential healthcare facilities increased from 17.8% (2011–2012) to 45.5% (2019–2020) [76]. Overall, uptake among hospital nursing staff was lowest among all staff categories for most seasons, but increased from 12.4% in 2011–2012% to 58.1% in 2019–2020 [76]. O'Lorcain et al. suggested that reasons for this improvement in seasonal influenza vaccine uptake were most likely associated with the development of strategic plans, and implementation of specific actions taken by the Health Service Executive and led and facilitated by staff, including occupational health, trained peer vaccinators, influenza vaccination mobilisers and management [76].

In a Danish study published in 2021, 51% of hospital healthcare professionals were reported to have received influenza vaccination, with formerly vaccinated participants more likely to be vaccinated again [77]. Perception of own gain, patient gain and a workplace recommendation were specified as key incentives for vaccine uptake [77].

High rates of vaccination coverage among healthcare professionals in hospitals in Finland were reported for 2017–2018 (83.7%), 2018–2019 (90.8%) and 2019–2020 (87.6%) [78]. The study authors proposed that the legal situation (semi‐mandatory system) in Finland, which defined that it was the employer's responsibility to appoint only vaccinated healthcare professionals to care for vulnerable patients, seemed to provide a good background for the high coverage rates during these seasons [78].

Table 1 shows a snapshot of current disparities in seasonal influenza vaccination in Europe.

**Table 1:** A snapshot of current disparities in seasonal influenza vaccination in Europe.

Disparity	Examples of disparity in seasonal influenza vaccination
Geographical disparity	Vaccination rates vary widely across Europe [6, 29]. In 2021, Ireland reported the highest rate in older populations (75.4%), with low rates in Slovakia (12.9%), Poland (10%), Bulgaria (8.9%) and Latvia (7.7%) [6].
Disparity between different ethnic groups	Research into disparities in influenza vaccination between different ethnic populations is primarily conducted in the USA [36, 37, 38, 39, 40]. A study in England showed that Black patients were least likely to be vaccinated (OR18–64 years: 0.82, and OR65+ years: 0.59) and Asian patients among 18–64 were most likely to be vaccinated (OR18–64 years: 1.10) [42] relative to White patients [42].
Socioeconomic disparity	Socioeconomic deprivation is correlated with low influenza vaccination coverage [42, 47, 48]. Higher vaccine uptake in individuals aged ≥ 60 years in Europe was reported for those not living alone (OR 1.39), with higher income (OR 1.26), and with higher education (OR 1.05) [45]. In the homeless population in England, vaccination levels are less than half those seen in the rest of the population [51].
Disparity related to sex and gender	In a study in Poland, males were more often vaccinated than females (OR 1.56). A study in England showed that females aged 18–64 years were more likely than males to be vaccinated (OR 1.19).
Disparity related to age	Vaccination rates are higher in older adults (65+ years), with rates around 74.0% in England [42]. In Poland, people aged over 65 years received influenza vaccination more often than younger counterparts (OR 1.64) [56]. Vaccination rates for children vary. In the Netherlands, GP consultation rates associated with influenza infection are highest in children under 5 years [59].
Disparity related to comorbidities	In a study in Denmark, only 59% of patients with rheumatoid arthritis receiving immunosuppressive drugs were vaccinated against influenza [70]. Only 10% of patients with multiple sclerosis were vaccinated against influenza in a study in Austria [71]. In a study in the Czech Republic, only 6.7% of patients with newly diagnosed HIV infection were vaccinated [72]. A further study in Denmark showed that influenza vaccination coverage among patients with heart failure ranged from 16% to 54% [74].
Disparity among healthcare professionals	Uptake of seasonal influenza vaccination among healthcare professionals varies considerably across Europe. In a cross‐sectional study in France, 34.8% of healthcare professionals were vaccinated in 2019 [75]. In Ireland, vaccination uptake among hospital healthcare professionals increased from 18.1% in 2011–2012% to 58.9% in 2019–2020 [76]. In a Danish study published in 2021, 51% of hospital healthcare professionals were vaccinated [77]. In Finland, vaccination coverage rates among hospital healthcare professionals were 83.7% (2017–2018), 90.8% (2018–2019), and 87.6% (2019–2020) [78].

GP, general practitioner; OR, odds ratio

### 1.2 Potential Reasons for Disparities in Seasonal Influenza Vaccination

Potential reasons for the disparities in seasonal influenza vaccination described above include knowledge and level of education, attitudes and behaviours towards vaccination, vaccine hesitancy and vaccine fatigue, and access to vaccination. These are described in the following sections.

#### 1.2.1 Knowledge, Level of Education and Seasonal Influenza Vaccination

Self‐reported data on influenza vaccination from 2019 show that higher proportions of individuals who completed tertiary education were vaccinated than those with a lower level of education in Hungary, Poland, Finland, Estonia and Norway, and the opposite was observed in Portugal, Belgium, Spain, Italy and Iceland [6]. Higher level of education was also consistently associated with higher coverage in a study in Norway reported in 2023 [79].

Higher level of education, being more knowledgeable about influenza vaccination, and taking more medications were independent predictors for being ever‐immunised against seasonal influenza in individuals aged over 65 years in Serbia [80]. In addition, older age, educational level, and information from, and recommendation by, a specialist or general physician were positively associated with influenza vaccine uptake in a study of patients with rheumatoid arthritis receiving immunosuppressive drugs in Denmark [70].

A total of 64.2% of pregnant women in a study in Italy were aware that influenza is more dangerous during pregnancy, yet only 9.7% had received the vaccine and 21.4% of those unvaccinated were willing to receive it [81]. Factors including older age, high‐risk pregnancy and tertiary education were associated with a higher likelihood of being knowledgeable about influenza vaccination [81]. The majority of respondents considered the vaccine not very useful during pregnancy [81]. In another Italian study, only 2.8% of pregnant women correctly identified all the vaccinations recommended during pregnancy and 1.4% had received seasonal influenza vaccination [82].

In a study of pregnant women in Germany, receiving a recommendation to vaccinate was strongly associated with influenza vaccine uptake, but only one‐fifth of women reported receiving such a recommendation, and uptake of influenza vaccine was only 13% [83]. Raising healthcare professionals' awareness of vaccinating during pregnancy is needed to increase vaccine uptake in pregnant women.

Health literacy is a key determinant of vaccine decision‐making [84]. A cross‐sectional study in Spain showed that pregnant women with high health literacy were more likely to decline influenza vaccination; 24% of women who declined felt the vaccine was unnecessary and 23% claimed to have insufficient information [85]. In contrast, health literacy did not affect the likelihood of influenza vaccine uptake among high‐risk groups in a study in Italy [86].

Less than half (42.3%) of participants in a study in Austria considered themselves to be sufficiently informed about national vaccination recommendations, with GPs the primary source of healthcare‐related information for the majority (73.1%) of participants [87].

A survey of 1106 adults with non‐communicable diseases in eight European countries showed that the main reasons for agreeing to have the influenza vaccine were disease prevention and healthcare practitioner recommendations [88]. Information provided to participants included how vaccines protect against infection, the risks of not getting vaccinated, possible side effects, duration of protection, and the need for a booster dose [88]. Unvaccinated individuals had received less information on these topics than those vaccinated [88]. A lack of belief in the need for an influenza vaccine, treating practitioners not recommending the vaccine, and experience of mild severity of influenza were the main barriers against the influenza vaccine [88]. The physician was the most preferred and tapped resource for information on influenza vaccines, followed by dedicated websites [88].

Considering these studies, differences in level of education and knowledge about influenza and influenza vaccination may contribute to the disparities in seasonal influenza vaccination in Europe. This observation provides a focus for reducing the disparity through a variety of educational and media initiatives.

#### 1.2.2 Attitudes and Behaviours Towards Seasonal Influenza Vaccination

A cross‐sectional survey in Greece showed that around half of eligible participants aged over 60 years received annual influenza vaccinations, and that behavioural (*p* < 0.001), normative (*p* < 0.001), and control (*p* < 0.001) beliefs and an increased intention score (*p* < 0.001) were associated with increased probability of vaccination [89]. In this context, these beliefs can be explained as follows. Behavioural beliefs include an individual's beliefs regarding the importance, necessity and safety of the influenza vaccination upon their own and their family's health [89]. In addition, normative beliefs refer to the influence of significant others (doctors, pharmacists, family, friends and other significant members of society) on an individual's behaviour and actions associated with influenza vaccination [89]. Lastly, control beliefs refer to an individual's beliefs about the presence of factors that may facilitate or hinder performance of a behaviour, in this case, the extent of control the individual has over the decision to get vaccinated [89].

Health behaviours, such as regular visits to the doctor, general health examinations and nonsmoking, have been reported to be significantly associated with higher odds of seasonal influenza vaccine uptake [64, 89].

Reasons for declining vaccination in a study of individuals aged over 65 years in Serbia included ‘they were in good health’ (33.5%) and ‘they did not believe that vaccine protects from flu’ (31.5%) [80]. Similarly, in a study in patients with rheumatoid arthritis in Denmark, reasons for not being vaccinated included fear of adverse effects, lack of information and recommendation, and perception of good health [70]. A total of 11.4% of participants in an Austrian cross‐sectional survey deliberately refused vaccinations (including for influenza) [87]. The most common reasons for non‐vaccination were fear of adverse effects (35.9%), doubt of effectiveness of vaccines (35.9%) and distrust of the pharmaceutical industry (23.1%) [87]. Lack of information, perception of own good health, and fear of adverse reactions were primary reasons for not being vaccinated among kidney transplant patients in a Danish study [73].

In a cross‐sectional questionnaire‐based study, only 4.5% of Polish teachers reported receiving influenza vaccine in the 2018–2019 season [90]. The main reasons for not being vaccinated were a lack of confidence in vaccine effectiveness (56.9%) and concerns related to adverse effects (30.6%) [90]. Although 43.8% of teachers believed that they were at risk of influenza infection, only 62.5% indicated vaccination as an effective method of preventing influenza [90]. This is an area that requires research as teachers are a potential target group for immunisation programmes against influenza infection [90].

In a 2009 study in a French teaching hospital, 22.3% of healthcare workers were vaccinated against seasonal influenza, with the immunisation coverage rate significantly higher among physicians [91]. The two most cited reasons for vaccination were ‘to protect the patient’ and ‘to avoid getting sick’; arguments against vaccination were ‘I never get the flu’ and ‘getting vaccinated is inconvenient and takes too long’ [91].

A study in Germany showed that the efficacy of seasonal influenza vaccination was misjudged by 77.8% of trainee midwives, possible adverse events were correctly estimated by only 35.2%, and 56.2% were not convinced of the safety of influenza vaccination during pregnancy [92]. Although 76.8% of trainees reported a positive attitude towards vaccinations in general, 73.3% complained of receiving too little information on complications due to vaccines [92].

Another example of inconsistent behaviour surrounding influenza vaccination is shown in a study in which a total of 51.5% of nurses and 90.6% of doctors in an Italian tertiary children's hospital believed that their risk of contracting influenza was greater than that of the general population, yet 77.8% of nurses and 45.8% of doctors were unvaccinated (*p* < 0.0001) [93].

Poor adherence to influenza vaccination guidelines by healthcare professionals, leading to low vaccination rates among their patients, has been reported in studies of community‐dwelling individuals with spinal cord injury in Switzerland (10% in those aged < 45 years) [94], and asplenic patients (59%) [95] and obese patients (32.2%) [96] in France. In addition, adherence to guidelines by GPs in France varies across regions [97]. Adherence to guidelines for pregnant women has also been reported as suboptimal in studies in France (47.9%) [96] and Greece (16.2%) [98]. In the latter study, healthcare professionals appeared to be reluctant to recommend vaccination during pregnancy [98]. Missed opportunities to vaccinate have been reported to be a primary reason for suboptimal seasonal influenza vaccination coverage in older adults in Italy [99].

As shown, attitudes and behaviours towards influenza and seasonal influenza vaccination clearly influence whether healthcare professionals recommend vaccination to their patients and whether patients choose to accept it. Strategies to reduce disparities in seasonal influenza vaccination across Europe should include consideration of this important topic.

#### 1.2.3 Vaccine Hesitancy and Vaccine Fatigue

Vaccine hesitancy refers to the refusal or delay in acceptance of vaccination despite the availability of vaccination services [100]. This hesitancy arises from reasons such as safety concerns about vaccination; beliefs that vaccines do not work, or that the risks of vaccination outweigh the benefits, and perceptions of low likelihood of contracting vaccine‐preventable diseases [101]. In 2019, before the COVID‐19 pandemic, vaccine hesitancy was considered by the WHO to be a top 10 threat to global health [102, 103]. Vaccine hesitancy is complex and is linked to human behaviours, including complacency, distrust, identity, beliefs and world views, and a fear of harm [103]. Understanding the reasons behind these behaviours is important when considering potential solutions that could lead to greater vaccine acceptance [103].

Vaccine hesitancy is common among healthcare professionals in Europe, necessitating shared strategies across all European countries to reduce disparities in this area [104]. Seventeen percent of primary healthcare workers in a study in Croatia were identified as vaccine hesitant [105]. Nurses and physicians differed (*p* < 0.001) in their general attitude, beliefs and behaviours towards vaccination, with nurses showing a higher level of hesitancy (adjusted OR 5.73) [105]. Personal hesitancy may negatively impact vaccination education and recommendations; therefore, interventions are needed to increase vaccination knowledge and confidence among primary healthcare workers, particularly nurses [105].

The COVID‐19 pandemic has changed attitudes towards vaccinations, and vaccine fatigue (inertia or inaction towards vaccine information or instruction due to perceived burden and burnout [106] has been a large contributor [107]. In a study of the changes in attitudes and barriers to seasonal influenza vaccination from 2007 to 2023 in university students in the USA, time, convenience, and perceived risk continued to be significant barriers to vaccination, and although the students were getting more encouragement to vaccinate from their healthcare providers and parents, this encouragement was becoming less effective [107]. The study authors considered that effective and empathetic vaccine communications are needed to eliminate preventable vaccine fatigue across sectors in society [107].

Uncertainty and misinformation surrounding vaccines is well recognised; however, the growing popularity of the antivaccine movement, fuelled by social media as a major contributor to vaccine misinformation, is creating disparity, impacting vaccination coverage and affecting global epidemiology [107, 108].

#### 1.2.4 Access to Vaccination

Influenza vaccination policies are vital to establishing a coordinated influenza vaccination programme, but do not guarantee equitable access or ensure vaccination coverage [109]. Reliable production of vaccine in relevant quantities and at an affordable price is critical for developing a successful vaccination policy; however, effective collaborations between private manufacturers, regulatory authorities and national and international public health services are also necessary to ensure optimal access and uptake [110].

National influenza vaccination policies vary by region, income, and immunisation system strength, and are less common in lower‐income countries [109]. A lack of healthcare infrastructure hinders the internal distribution of and access to vaccines, particularly in remote communities, where motivating people to visit vaccination centres located far away may not be successful [111]. Barriers to adoption of influenza vaccination policy, such as cost, logistics, country priorities, need for yearly vaccination, and variations in seasonality, need to be addressed [109].

The relatively low level of influenza vaccination coverage in most European countries is largely due to patients having limited access to vaccinations [112]. All 120 maternity trusts included in a study in England were commissioned to deliver influenza vaccinations, although not all actually administered the vaccines and there was large between‐trust variation in vaccination rates [113]. Reasons for these variations should be investigated to improve uptake and equity [113].

Currently, 15 European countries allow community pharmacists to dispense and administer influenza vaccines [114]. Therefore, this represents another disparity across Europe as many countries have not implemented the vaccinations in pharmacies. In some countries, implementing vaccinations in community pharmacies and by authorised pharmacists has significantly improved vaccination coverage rates and herd immunity [112].

Influenza co‐infection was associated with severity and mortality in patients with COVID‐19 during the pandemic [115]. Increased coverage of influenza vaccination was encouraged throughout this period to mitigate the transmission of influenza virus and reduce the risk of severe outcome and mortality [115]. Access to influenza vaccines, as well as COVID‐19 vaccines, was a priority alongside COVID‐19 testing and public health and social measures [116]. Efforts to increase influenza vaccine uptake during a respiratory virus pandemic should be consolidated in future clinical practice [117].

Access to vaccine is a crucial aspect of effective seasonal influenza vaccination programmes and is a key area to address to reduce disparity in vaccine uptake.

### 1.3 Addressing Disparities in Seasonal Influenza Vaccination in Europe

Primary care practitioners have an important role in informing patients about vaccines and healthcare topics; therefore, strengthening the role of GPs in promoting influenza vaccination, including highlighting the personal and community benefits, is fundamental to reducing disparities in influenza vaccination and improving vaccination rates across Europe [47, 87].

Enhanced knowledge about influenza has a positive impact on decisions about vaccination; therefore, the focus should be on education strategies that reduce knowledge gaps, with the aim to improve attitudes, address disparities, and increase vaccination rates [90]. ‘Tailor‐made’ information campaigns to promote health and vaccination that take into account the needs of the elderly, individuals with comorbidity, or other specific patient groups, are essential to reduce disparities and improve influenza vaccination coverage rates in these populations [47, 66, 70, 95]. Health education programmes are also needed across Europe to improve the understanding of healthcare professionals and pregnant women about seasonal influenza and vaccination in pregnancy [81]. In addition, quantification of links between social factors and lower vaccination rates could empower healthcare professionals to target particular social groups in the quest to address vaccine‐related inequalities [45]. Information campaigns stressing the necessity for healthcare workers to be vaccinated must also be strengthened [91].

Healthcare practitioners are key advocates for influenza vaccination, and should be part of an increase in the dissemination of information about the importance of influenza vaccine, with incisive messaging based on local barriers an important strategy for increasing vaccine uptake [88]. Efforts to improve the education and knowledge gap associated with influenza vaccination should include tackling the broad spectrum of false contraindications in clinical practice, which directly impact on vaccination coverage [118, 119, 120].

Concerns about safety, the possibility of side effects and the vaccine development process need to be addressed [91, 121]. Utilising debunking in media campaigns as well as vaccine information and social norm modelling should help to dispel misinformation and address distrust associated with vaccination, and optimise the acceptance of vaccines in different populations [122].

It is essential to improve understanding of the main reasons for patients' vaccine hesitancy based on specific sociocultural, behavioural and psychological factors [103]. This knowledge may help guide healthcare professionals to recognise vaccine hesitancy and address patients' cognitive and behavioural biases, thereby enabling them to instigate meaningful conversations with patients and/or their caregivers, and develop more personalised solutions to improve acceptance of vaccinations [103].

Broadening the range of settings through which individuals can receive influenza vaccination, including children of school age through their schools [123]; adults through pharmacies [124]; pregnant women through their midwives [125]; and healthcare workers through their workplace [8], is a further strategy that could reduce disparity in influenza vaccination and boost vaccination coverage. Options to expand the role of the pharmacist in the provision of immunisation services for vaccine‐preventable diseases include improving communication with healthcare policymakers and the public, generating real‐world evidence that highlights the public health benefits of vaccination, and providing continuing professional education and training for pharmacists [114].

The use of less invasive, intranasal influenza vaccine might reduce the fear and discomfort associated with vaccine injections in certain subjects, thereby potentially improving adherence to vaccination. Also important is structured counselling on immunisation that provides accurate, evidence‐based information that dispels myths, addresses misbeliefs, and tackles prejudice, and negative attitudes towards vaccination [80]. Increased awareness about guidelines and providing educational resources for physicians are also warranted [73].

Improved understanding of the wider impact of influenza can support health authorities in their decisions surrounding vaccination programmes [126]. Addressing known disparities in influenza vaccination and increasing vaccination coverage to reach the WHO target of 75% across Europe requires an integrated strategy that strengthens awareness among healthcare policymakers and the public, and inspires healthcare workers to become proactive vaccination advocates [126].

Table 2 shows selected disparities and suggested stakeholders or ‘problem owners’ who could take responsibility for specific disparities. Also presented in the table are proposed actions that specific stakeholders could take to solve a particular defined sub‐issue within the complex, multifactorial disparities in influenza vaccination. The aim of this table is to stimulate discussion among stakeholders on how to tackle these important public health disparities, to highlight the importance of assigning stakeholders to take ownership of particular issues, and to support stakeholders who are struggling with these issues worldwide.

**Table 2:** Suggested stakeholders and proposed actions for specific disparities.

Disparity	Stakeholder(s)	Proposed action
Geographical disparity in seasonal influenza vaccination	National and local health authorities, policymakers	Implement targeted vaccination campaigns in regions with low coverage, improve data collection and monitoring systems
Disparity in seasonal influenza vaccination between different ethnic groups	Healthcare providers, community leaders, policymakers	Develop culturally sensitive education and outreach programmes, build trust in healthcare systems, address institutional and systemic racism
Socioeconomic disparity in seasonal influenza vaccination	Social services, healthcare providers, policymakers	Design interventions that address socioeconomic barriers, provide free or subsidised vaccines, outreach to vulnerable populations
Disparities in seasonal influenza vaccine uptake related to sex and gender	Healthcare providers, policymakers	Incorporate sex‐specific strategies in vaccination campaigns, conduct research on differences in vaccine uptake between males and females
Disparities in seasonal influenza vaccine uptake related to age	Healthcare providers, schools, policymakers	Expand vaccination programmes to include younger age groups, promote vaccination among working‐age adults, inform older adults of the importance of vaccination
Disparities in seasonal influenza vaccine uptake related to comorbidities	Healthcare providers, patient advocacy groups, policymakers	Raise awareness about the importance of vaccination for those with comorbidities, ensure easy access to vaccines
Disparities in seasonal influenza vaccination among healthcare professionals	Healthcare institutions, professional organisations, policymakers, medical schools, training providers	Increase vaccination promotion among healthcare professionals, provide incentives and education, include more comprehensive teaching on immunisation and vaccination in curricula for healthcare students
Vaccine hesitancy and vaccine fatigue	Healthcare providers, policymakers, media	Address misinformation and build trust through transparent communication, use social norm modelling
Access to vaccination	National and local health authorities, policymakers, healthcare providers, immunisation programme managers, pharmacies	Enhance vaccine availability through pharmacies and community centres, ensure equitable distribution

## 2 Conclusions

Addressing disparities in influenza vaccination in Europe will improve vaccination coverage and help reduce influenza‐associated morbidity, hospitalisations, and mortality, thus reducing the burden of influenza on individuals and healthcare systems. Widespread and consistent implementation of influenza vaccination strategies that include the working‐age population will also reduce absenteeism from work and the associated impact on productivity as well as local and national economies.

Recognising differences in perception and uptake of influenza vaccination for children *vs.* that for adults might enable a more comprehensive approach to promote vaccination ‘as a whole’ to prevent disease, and could provide some insight into how to increase interest in adult vaccinations. Engaging adults to commit to vaccination requires understanding of the complex human decision‐making process based on perception of risk (of the disease and the vaccine) and the effort required to have vaccination annually, and the convoluted decision process surrounding influenza vaccination.

Understanding disparities in influenza vaccination may also shed some light on disparities associated with other types of vaccination. In addition, proposed solutions to expand influenza vaccination may also be useful for other types of vaccination and vice versa.

Preventive strategies such as vaccination are fundamental to promote healthy ageing, which is particularly relevant considering the ageing population in Europe and the associated increasing burden placed on healthcare and economic resources.

## Author Contributions

**Katinka M. Giezeman‐Smits:** conceptualisation, data curation, investigation, methodology, resources, supervision, validation, writing – original draft, writing – original draft. **Bram Palache, Gerrit A. van Essen, Miloš Jeseňák, Enrique Castro‐Sánchez, Anna Elisabeth Steinberg:** conceptualisation, writing – review and editing. **Joris van Vugt:** conceptualisation, data curation, investigation, methodology, resources, supervision, validation, writing – original draft, project administration, supervision, validation.

## Ethics Statement

The authors have nothing to report.

## Consent

The authors have nothing to report.

## Conflicts of Interest

BP received consultancy fees from Abbott, Viatris, and Osivax. Member of Vaccine Europe, and IFPMA. He is cofounder of and advisor for ESWI. GAvE received speakers fees from GSK, MSD, Moderna, Pfizer, Sanofi and Viatris. MJ received speaker fees from GSK, MSD, Moderna, Pfizer, Sanofi, Swixx Pharma and Viatris. KMGS, AES, JvV, ECS have no disclosures.

## Data Availability

All data generated or analysed during this study are included in this published article.
